# Nucleation of Frank Dislocation during the Squeeze-Out Process in Boundary Lubrication: A Molecular Dynamics Study

**DOI:** 10.3390/ma15030997

**Published:** 2022-01-27

**Authors:** Rong-Guang Xu, Yuan Xiang, Gunan Zhang, Qi Rao, Yongsheng Leng

**Affiliations:** Department of Mechanical and Aerospace Engineering, The George Washington University, Washington, DC 20052, USA; xyuan@gwmail.gwu.edu (Y.X.); gunan@gwmail.gwu.edu (G.Z.); rao@gwmail.gwu.edu (Q.R.)

**Keywords:** lubrication, nanotribology, Frank dislocation, phase transition, molecular dynamics

## Abstract

Liquid–vapor molecular dynamics (LVMD) simulations are performed to reinvestigate the phase transition and solvation force oscillation behavior of a simple argon liquid film confined between two solid surfaces. Our simulations present a novel scenario in which the n → n − 1 layering transitions are accompanied by the formation, climb, and annihilation of Frank partial dislocations during the squeeze-out process under compression. This is indicated by the splitting of the repulsive peaks in the solvation force profile. The detailed analysis reveals that the formation–climb–annihilation mechanism of Frank dislocation occurs during approach and disappears during receding, which would result in force hysteresis. In combination with our recent works, this study provides new insights into the physical property of nanoconfined lubricant films in boundary lubrication.

## 1. Introduction

Nanometer-thick liquid film confined between two solid surfaces has fundamental implications for surface and interfacial science [1]. Frictional and lubrication properties of a film in a confined geometry are affected by the way it reacts to external loading (such as compression and shear) [2,3,4,5]. A long-standing debate in surface force experiments has been the structure and shear properties of simple nonpolar lubricants confined between two solid surfaces [6,7,8,9,10,11,12,13,14,15]. One critical question is that under normal compression between two molecularly smooth surfaces, how does a nonpolar lubricant film proceed in a thinning or squeeze-out process? As a result of this process, what is the molecular packing structure of the film? This is crucial in the design of preventative strategies against friction and surface failure during tribological loading.

Recently, we developed and validated a liquid–vapor molecular dynamics (LVMD) simulation method [16,17,18] to investigate the phase behaviors and shear properties of spherical molecular liquids. The most important finding is that cyclohexane (C_6_H_12_), a widely used simple model lubricant studied in surface force apparatus/balance (SFA/B) experiments, underwent a sudden liquid-like to solid-like phase transition at n < 6 monolayer thickness when confined between two molecularly smooth mica surfaces [19]. The simulated force–distance profiles are remarkably consistent with Jacob Klein′s SFB experimental results [6,7]. LVMD friction simulations showed that shearing of solidified cyclohexane film at three- or four-monolayer thickness resulted in stick–slip friction. We found that instead of shear melting of the film during the slip of the surface [4], boundary slips at the solid–lubricant interface occurred, while the solidified structure of the lubricant film was well maintained during repeated stick–slip friction cycles.

The nature of nucleation and propagation of partial dislocations play an essential role in the evolution of microstructure and mechanical properties of crystalline materials in nanoscales such as twinning reactions, phase transformations, and the formation of dislocation barriers by intersecting dislocations [20,21]. In face-centered cubic (FCC) metals, there are two important types of partial dislocations: Shockley partial dislocations and Frank partial dislocations. Since Shockley partial dislocations dominate the dislocation avalanche process when yielding occurs under external loading, the characteristics of Shockley partial dislocations linked to plastic deformation of FCC metals have been widely researched [22,23,24,25]. Movement-wise, Shockley partials are glissile, whereas Frank partials are sessile. Shockley partial dislocation is associated with slip on the {111} slip planes since its Burgers vector lies parallel to the slip plane. On the other hand, Frank partial dislocations outline a stacking fault formed by inserting or removing a region of closed packed {111} layer of atoms, which is referred to as extrinsic (positive Frank partial) or intrinsic (negative Frank partial) stacking fault, respectively. A Frank dislocation is a pure edge dislocation, and its Burgers vector can be expressed as $\;b\;\to =\frac{1}{3}\langle 111\rangle$, which is perpendicular to the stacking faults layer. Frank partial dislocations are classified into “sessile dislocations” since they rarely propagate and therefore are seen as a “signature” reflecting microscopic processes due to their stability and immobility. Positive Frank dislocations are usually produced by the precipitation of interstitial atoms as a result of radiation damage. In contrast, negative Frank dislocations occur when a platelet of vacancies collapses due to the local supersaturation of vacancies caused by rapid quenching or from the displacement cascades generated by irradiation with energetic atomic particles [21]. As compared with Shockley partial dislocation, Frank partial dislocation has been less studied.

Our recent study showed that in stick–slip friction in boundary lubrication with commensurate contact between the confining wall and the solidified film, interlayer or boundary slips are always accompanied by the nucleation–propagation–annihilation process of Shockley partial dislocations [26]. Because shear-induced Shockley partial dislocations can be observed, a fascinating question would be whether compression-induced Frank partial dislocations can also be observed in the same system. The duality of shear-induced Shockley partial dislocations and compress-induced Frank partial dislocations might play a crucial role in determining the mechanical properties of confined thin films and thus open up new research avenues.

Moreover, our previous study [18] showed that the solvation force hysteresis observed in LVMD simulations between the normal approach and receding curves, as seen frequently in many SFA/B experiments, is due to two principal effects. The first is connected to the unstable jumps that occur during the laying transitions of the confined film, where the force gradient is greater than the driving spring constant. The second effect is related to the squeeze-out dynamics of the confined film, even if the first effect is absent. In general, these two dynamic processes are non-equilibrium in nature and involve significant energy dissipations, resulting in force hysteresis. As discussed in this study, a significant aspect of the force–distance profiles obtained from the LVMD simulation is related to the asymmetric oscillations of solvation forces, i.e., the repulsive peaks are always greater than the adhesion valleys. This interesting phenomenon was observed in many SFA/B experiments. An explanation of the asymmetric force oscillation behavior can be traced to the different compressive and tensile properties of the solidified film. A significant feature of this simulation is that, upon receding, the repulsive forces peaks cannot reach those experienced in a normal approach, resulting in force hysteresis. The detailed structural analysis further demonstrates that the solidified structure upon receding fails to return to its original compressive state during the normal approach, i.e., more atomic defects in the receding structure than in approach one, which explains the origin of the force hysteresis.

In this study, our simulations present a new scenario in which Frank partial dislocations can nucleate, climb and annihilate during n → n − 1 layering transition under compression. However, we did not observe such a phenomenon in n → n + 1 layering transition during receding. In combination with our recent works, these observations can add a new physical mechanism to account for the solvation force hysteresis. This study provides new insights into the physical property of nanoconfined lubricant films in boundary lubrication.

## 2. Computational Models and Simulation Methods

**Figure 1:**
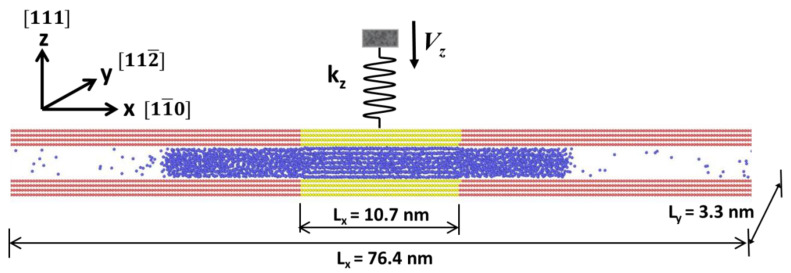
Schematic of the liquid-vapor molecular dynamics (LVMD) simulation method with a snapshot of the equilibrium configuration of an argon droplet confined between two solid walls. The central walls are in yellow, and the side walls are in red. The top wall is pushed by a spring connected to a stage that moves at constant velocity *V_z_*.

The computational models and simulation methods are the same as the case of commensurate contact discussed in our previous studies [16,17,18,26,27], which is briefly summarized here. Figure 1 illustrates an argon film, which serves as a nonpolar lubricant, confined between two rigid face-centered cubic (FCC) solid walls. As stated in the literature [28], we use a simple Lennard–Jones (LJ) pair potential to model the interaction between argon molecules, i.e., σ = 0.3405 nm and ε = 0.2381 kcal/mol. The two FCC crystal walls have the z-direction along {111} direction, while the x- and y-directions are along $\left\{1\bar{1}0\right\}$ and $\left\{11\bar{2}\right\}$ directions, respectively. Each confining walls consist of a central wall sandwiched by two side walls. These two regions have different arrangements of wall–fluid interactions. In the central region, the wall–fluid interaction between argon and the solid wall is the same as the argon–argon interaction (i.e., ε_wf_ = ε_ff_ = ε, where the subscript w refers to “wall” and f refers to “fluid” respectively). The wall–fluid interaction strength in the two side regions is reduced to one-fourth ($\frac{1}{4}$ε) that of the argon–argon interaction strength so that argon molecules remain in a liquid state. The two solid walls are subjected to periodic boundary conditions (PBCs) along the x- and y-directions. The dimensions of the solid central walls in the three dimensions are 10.7 × 3.3 × 0.9 nm^3^. Including the frictionless LJ walls, the total length of the simulation box in the x-direction is 76.4 nm. A standard cutoff distance of 8.5 Å (about 2.5 σ) is used for the LJ interactions, and the time step is 1.0 fs in MD simulations. All the simulations are performed with the Nosé–Hoover thermostat [29,30] at a temperature of 85 K, corresponding to a liquid state of argon (between the melting point (83.8 K) and the boiling point (87.3 K) of argon).

As shown in Figure 1, a liquid–vapor molecular dynamics (LVMD) ensemble was applied to the condensed liquid film during compression in order to allow it to squeeze out. This is achieved by extending the side walls in the squeeze-out direction. It is estimated that the vapor pressure is around a few atm [16,26], which is vanishingly low in comparison to the normal repulsive pressure on the solid phase. This method is particularly useful for the simulation of solvation force under normal compression, as well as for the sliding friction simulation of confined liquid systems where particle exchanges can occur between confined and bulk liquid phases. The LVMD method was applied successfully in our previous studies to investigate the mechanical properties of confined liquid films both in SFA/B [16,17,18,19,26] and AFM (atomic force microscopy) [31,32,33]. As the normal force in the surface force balance is measured through the compression of a normal spring, a driving spring-block system with force constants (k_x_, k_y_, k_z_) was used in simulations. Here, we selected k_x_ = 300 N/m, k_y_ = 300 N/m, and k_z_ = 150 N/m, which are consistent with SFA/B experiments [6,7] and our previous studies. The compression and receding rate in the LVMD simulation were at 0.05 m/s, which is the same as our previous studies.

The OVITO visualization package [34] facilitates the visual analysis of the local structural environment of atoms using the Polyhedral Template Matching (PTM) method [35] and the monitoring of dislocation movement using the Dislocation Extraction Algorithm (DXA) [36,37]. The PTM method is capable of identifying local crystalline structures of simple condensed phases formed by atoms ranging from some types of FCCs (face-centered cubic), HCPs (hexagonal close-packed), BCCs (body-centered cubics), SCs (simple cubics), and ICOs (icosahedrals) to others (unknown coordination structures). Even in the presence of strong thermal fluctuations and strains, it still performs effectively. The DXA can detect and analyze dislocations (including partial dislocations) in crystals, determine their Burgers vectors, and display a line-based representation of the dislocation network.

## 3. Results and Discussions

**Figure 2:**
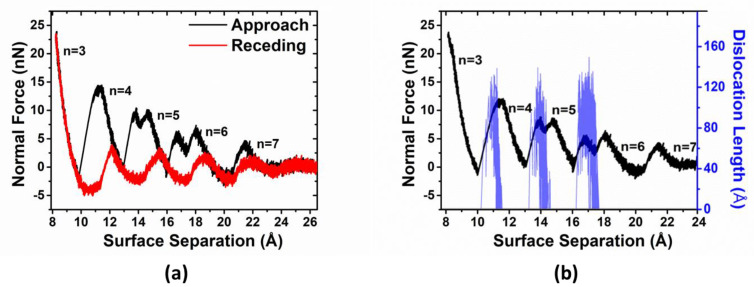
(**a**) Normal forces vs. surface separation between two solid surfaces across argon during approach (the solid black line) and receding (the solid red line). (**b**) The total length of Frank partial dislocation lines vs. surface separation during the normal squeeze-out process. The approach force profile is also shown to highlight the coincidence between the drop in force and the formation of Frank partial dislocations.

Figure 2a depicts the normal approach and receding force–distance profiles at a normal driving speed of v=0.05 m/s. In this commensurate contact, the critical layer number at which the liquid-like to solid-like phase transition happens is $n_{c}=7$, which is associated with significant repulsive solvation force. In contrast with previous studies [16,18], a double peak (or peak splitting) was observed in the 6 → 5 and 5 → 4 layering transitions in the approach simulations, indicating the presence of a new phenomenon. Such behavior is absent in the receding runs. It should be noted that in previous SFA/B experiments [6,7,10,12,38], instead of double peaks (or peak splitting), oscillatory profiles with single peaks were often observed in the measured solvation force.

In Figure 2b, we plot the variation of a total length of Frank partial dislocations, as well as the compression force as a function of the wall separation during the normal approach. We can see that during the 6 → 5 and 5 → 4 layering transitions, the second rise in normal force is associated with the formation of Frank partial dislocations right after the first force drop. They can maintain their stability until the second force drop, which annihilates upon the completion of the 6 → 5 and 5 → 4 layering transitions. It can still be observed that a formation–propagation–annihilation process of Frank dislocation occurs during the 4 → 3 layering transition, even if there is only one peak in normal force.

**Figure 3:**
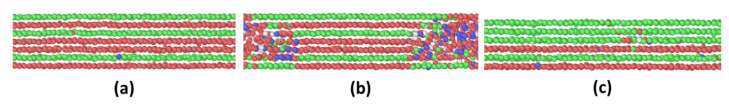
The dynamic progression of the 7 → 6 squeeze-out process of argon atoms confined between two solid walls. The packing structures are shown in (**a**) highly ordered 7-layer solid phase, (**b**) inward shrinking of the 7-layer solid phase, and (**c**) highly ordered 6-layer solid phase. Only atoms in the central confinement regime are shown. Atoms in face-centered cubic (FCC), hexagonal close-packed (HCP), body-centered cubic (BCC), and other mixed crystalline structures are in green, red, purple, and white, respectively. The color scheme is consistent throughout this work.

In our previous studies [16,18], the outward spreading of the solid phase during the liquid-to-solid phase transition and the inward shrinking of the solid phase during the solid-to-liquid phase transition are two universal phenomena observed in the LVMD simulations. However, those studies have a limited scope because the translational order parameter ρ(k) was used to characterize the crystalline structure. For a perfect lattice, ρ(k) is of order unity, whereas for liquid or disordered phase, it decays close to zero. Thus, it can differentiate solids from liquids, but not between HCP and FCC solid phases. In the following, we detect the detailed structural evolution of layering transitions under the squeezing process, starting with the highly ordered 7-layer solid phase, which is shown in Figure 3a. The ordered seven-monolayer structure has a mixed structure with three FCC and four HCP layers. The force decay corresponds to an inward shrinking of the solid phase, as shown in Figure 3b. This inward squeeze-out, initiated from the solid–liquid phase boundary, leads to complete melting of solid phase, followed by the recrystallization of a six-layer solid structure as shown in Figure 3c, which is the same as previous observations. 

**Figure 4:**
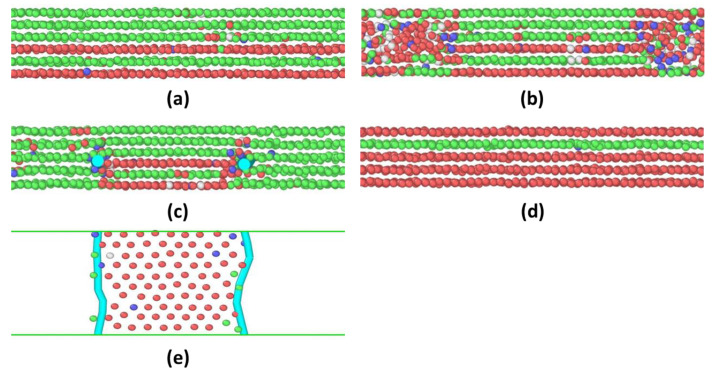
The dynamic progression of the 6 → 5 squeeze-out process of argon atoms confined between two solid walls. The packing structures are shown in (**a**) highly ordered 6-layer solid phase, (**b**) inward shrinking of the 6-layer solid phase, (**c**) 5layer–6layer–5layer solid phase, and (**d**) highly ordered 5-layer solid phase. Only atoms in the central confinement regime are shown. The two cyan dots in (**c**) and two cyan lines in the top-view configurations in (**e**) represent Frank partial dislocations.

At n = 6, as the gap distance decreases with increasing compression force, more atoms at both solid–liquid interfaces melt, corresponding to the first force decay (Figure 4b). Rather than continual shrinkage of the solid phase due to melting, previous melt atoms would recrystallize instead to create a sTable 5layer–6layer–5layer structure, as shown in Figure 4c. At the boundary of the five-layers and six-layers, two Frank partial dislocations are identified, as shown in Figure 4c,e. Geometrically, the current configuration of the confined film is equivalent to the insertion of one close-packed layer of atoms, which produces the extrinsic stacking fault. Generally speaking, inserting or removing one close-packed {111} layer of atoms can form a Frank partial dislocation by marking the boundary line of a fault. Its Burg’s vector is perpendicular to the dislocation line. An additional compression force (the normal force arises again) is needed to make these two Frank partial dislocations move inward to eliminate the extra {111} layer. This process is called “the climb” of Frank partials. The entire process takes approximately 2.4 ns.

**Figure 5:**
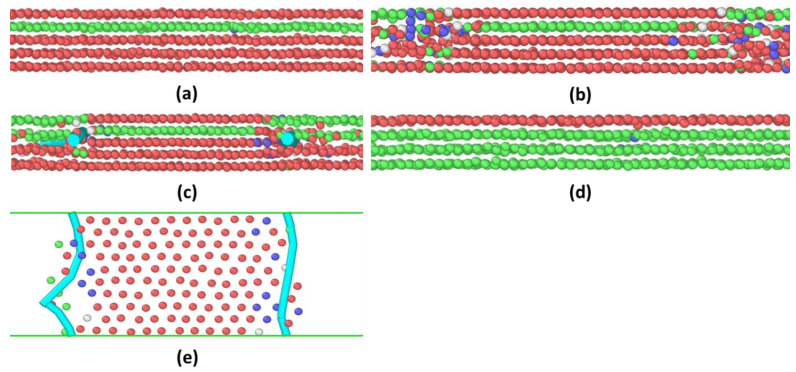
The dynamic progression of the 5 → 4 squeeze-out process of argon atoms confined between two solid walls. The packing structures are shown in (**a**) highly ordered 5-layer solid phase, (**b**) inward shrinking of the 5-layer solid phase, (**c**) 4layer–5layer–4layer solid phase, and (**d**) highly ordered 4-layer solid phase. Only atoms in the central confinement regime are shown. The two cyan dots in (**c**) and two cyan lines in the top-view configurations in (**e**) represent Frank partial dislocations.

Regarding the 5 → 4 layering transitions, the entire process follows the same pattern as the 6 → 5 layering transitions, as shown in Figure 5. The values of both peaks and the valley in between are larger than those during the 6 → 5 layering transitions (see Figure 2), indicating that it needs a larger force to drive the climbing process of Frank partials. It should be noted that the gap between two peaks and the peak-to-valley amplitudes are both diminished, compared with the 6 → 5 layering transitions. The entire process takes about 1.6 ns.

**Figure 6:**
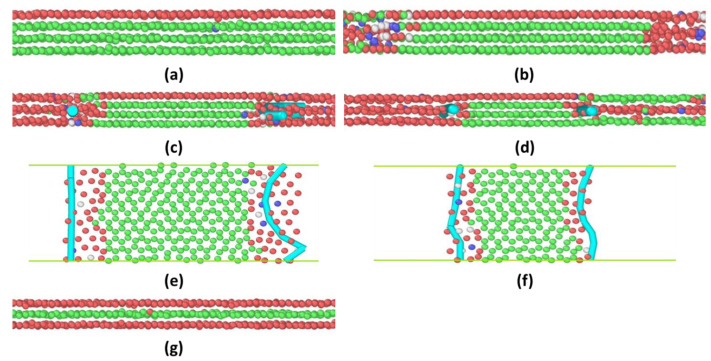
The dynamic progression of the 4 → 3 squeeze-out process of argon atoms confined between two solid walls. The packing structures are shown in (**a**) highly ordered 4-layer solid phase, (**b**) inward shrinking of the 4-layer solid phase, (**c**) 3layer–4layer–3layer solid phase, (**d**) inward shrinking of the 4-layer solid phase with inward growth of the 3-layer solid phase, and (**g**) highly ordered 3-layer solid phase. Only atoms in the central confinement regime are shown. The two cyan dots in (**c**,**d**) and two cyan lines in the top-view configurations in (**e**,**f**) represent Frank partial dislocations.

As shown in Figure 6, the entire process of the 4 → 3 layering transitions still follows the same procedure as the 6 → 5 and 5 → 4 layering transitions. However, a single peak in normal force is observed instead. In this case, it is expected that as the monolayer thickness decreases and the solvation force increases (the squeeze-out process becomes harder), the gap between two peaks and peak-to-valley amplitudes will shrink, and the two peaks will merge to form a single peak. Such a degeneracy of two peaks also indicates that the nucleation and climb of Frank partial dislocations do not need extra force to trigger, and the lifetime of the 3layer-4layer–3layer configuration (which is about 0.6 ns) is much shorter than that of the 5layer–6layer–5layer (2.4 ns) and 4layer–5layer–5layer (1.6 ns) ones in the 6 → 5 and 5 → 4 layering transitions. 

It should be noted that in Figure 2b, Frank dislocation lines can have a total length greater than 2 L_y_ (L_y_ with 3.3 nm width is the size of the simulation cell in y-dimension), which indicates that the Frank dislocation lines identified by the DXA algorithm are not straight as shown in Figure 4e, Figure 5e and Figure 6e,f. We found that the output length of dislocations depends heavily on the selection of two key parameters in the DXA process: the trial circuit length and the circuit stretchability, i.e., varying these parameters will affect the length of dislocations quantitatively, which, however, does not affect the conclusion of this study.

In the case of retraction loading, all the observations are consistent with previous studies [16,18], and no Frank dislocation is detected during the n → n + 1 layering transitions. Our previous study [18] revealed that the solvation force hysteresis commonly observed in SFA/B experiments and LVMD simulations between the normal approach and retraction loading is due to two principal effects: (1) the unstable jumps that occur during the layering transitions of the confined film, where the force gradient is greater than the driving spring constant. (2) the squeeze-out dynamics of the confined film. In general, these two dynamic processes are non-equilibrium in nature and involve significant energy dissipations, resulting in force hysteresis. The current work moves a further step forward and provides a more detailed picture of the structural evolution of confined film in the unstable jumps and the squeeze-out dynamics during the layering transitions. 

## 4. Conclusions 

In summary, our MD simulations of a simple argon liquid film confined between two solid surfaces reveal a previously unknown scenario, in which the n → n − 1 layering transitions are accompanied by the formation–propagation–annihilation process of Frank partial dislocations during the squeeze-out process under compression. In contrast, such a phenomenon was not observed in n → n + 1 layering transitions during receding. According to these observations, the solvation force hysteresis can be ascribed to this new physical mechanism. 

A major finding of this study is that the force–distance profile’s double peaks (or peak splitting) in the 6 → 5 and 5 → 4 layering transitions are emblematic of the nucleation of Frank partial dislocations. However, in previous SFA/B experiments [6,7,10,12,38], instead of double peaks (or peak splitting), oscillatory force profiles with single peaks were often observed. As it is well-known, there has been a long-term controversial debate on the layering transition (i.e., liquid-like to solid-like phase transition versus amorphous glass-like transition) of lubricants during a squeeze-out process under compression. The oscillatory force profile alone cannot indicate the nature of the layering transitions. Nevertheless, if a future SFA/B experiment can capture the peak splitting in repulsive force peaks in confined thin films, strong evidence of a first-order solidification accompanied by the nucleation of Frank partial dislocations could be confirmed. This mechanism cannot be excluded, however, even if only single peaks in solvation forces are observed, as in the case of the 4 → 3 layering transitions in this study.

Because the nature of solidification is stochastic, the crystalline structure during the confinement-induced liquid-to-solid phase transition can adopt different crystalline structures, i.e., different amounts of a mixture of FCC and HCP crystalline structures. As such, different oscillatory force profiles with single peaks or double peaks can be observed, depending on the nature of the dynamical evolution of crystalline configuration. Once the contact area becomes very large (about one order magnitude larger than the one in this study (10.7 × 3.3 nm)), the configuration of a solidified film can be very complicated, and the polycrystalline structures would form [26]. Furthermore, a variety of other factors can also affect the phase behavior of the confinement-induced phase transition: (1) the stacking sequences of two confining surfaces, (2) the relative crystallographic configurations or the misfit angle between two confining surfaces, (3) the roughness of the confining surfaces, and (4) the driving speed of the normal spring. The relevant physical and mechanical properties such as solvation force, nucleation rate of the liquid-to-solid phase transition, the phase behavior during the phase transition, and dynamics of the partial dislocations in the confined solids are all rate-dependent, i.e., they heavily depend on the driving speed of the normal spring. The effect of these factors will be under investigation in our future studies, which would significantly enhance our fundamental understanding of the mechanical properties of nanoconfined liquid films, especially the mechanisms of phase transition and interlayer/boundary slips of simple model fluids.

## Data Availability

All the data is available within the manuscript.
